# Secretory SERPINE1 Expression Is Increased by Antiplatelet Therapy, Inducing MMP1 Expression and Increasing Colon Cancer Metastasis

**DOI:** 10.3390/ijms23179596

**Published:** 2022-08-24

**Authors:** Won-Tae Kim, Jeong-Yeon Mun, Seung-Woo Baek, Min-Hye Kim, Gi-Eun Yang, Mi-So Jeong, Sun Young Choi, Jin-Yeong Han, Moo Hyun Kim, Sun-Hee Leem

**Affiliations:** 1 Department of Biomedical Sciences, Dong-A University, Busan 49315, Korea; 2 Metabolic Regulation Research Center, Korea Research Institute of Bioscience and Biotechnology (KRIBB), Daejeon 34141, Korea; 3 Department of Bioinformatics, KRIBB School of Bioscience, Korea University of Science and Technology, Daejeon 34113, Korea; 4 Department of Health Sciences, The Graduated of Dong-A University, Busan 49315, Korea; 5 Department of Cardiology, Dong-A University Hospital, Busan 49201, Korea; 6 Department of Biomedical Laboratory Science, Daegu Health College, Daegu 41453, Korea; 7 Department of Laboratory Medicine, Dong-A University Hospital, Busan 49201, Korea

**Keywords:** SERPINE1, MMP1, cancer metastasis, antiplatelet agents

## Abstract

Contrary to many reports that antiplatelet agents inhibit cancer growth and metastasis, new solid tumors have been reported in patients receiving long-term antiplatelet therapy. We investigated the effects of these agents directly on cancer cells in the absence of platelets to mimic the effects of long-term therapy. When four antiplatelet agents (aspirin, clopidogrel, prasugrel, and ticagrelor) were administered to colon cancer cells, cancer cell proliferation was inhibited similarly to a previous study. However, surprisingly, when cells were treated with a purinergic P2Y12 inhibitor (purinergic antiplatelet agent), the motility of the cancer cells was significantly increased. Therefore, gene expression profiles were identified to investigate the effect of P2Y12 inhibitors on cell mobility, and Serpin family 1 (SERPINE1) was identified as a common gene associated with cell migration and cell death in three groups. Antiplatelet treatment increased the level of SERPINE1 in cancer cells and also promoted the secretion of SERPINE1 into the medium. Increased SERPINE1 was found to induce MMP1 and, thus, increase cell motility. In addition, an increase in SERPINE1 was confirmed using the serum of patients who received these antiplatelet drugs. With these results, we propose that SERPINE1 could be used as a new target gene to prevent the onset and metastasis of cancer in patients with long-term antiplatelet therapy.

## 1. Introduction 

Platelets, which are present in blood vessels, are known to influence a variety of mechanisms, including hemostasis, vascular stabilization, and inflammatory responses [1,2,3]. Activated platelets increase adhesion and release various factors that allow aggregate formation by binding to leukocytes and cancer cells in the blood vessels [1,4]. Furthermore, activated platelets interfere with the immune response induced by natural killer cells, protecting circulating tumor cells in the blood vessels from immune elimination and attaching to the vascular endothelium to promote metastasis [1,5]. Therefore, activation of platelets and clotting systems plays an important role in cancer progression and is also known to be associated with cancer metastasis [1,2,3,6].

Numerous medications inhibit platelet function, including aspirin, phosphodiesterase inhibitors, purinergic P2Y12 inhibitors, glycoprotein (GP) IIb/IIIa antagonists, integrin (αvβ3) antagonists, thromboxane synthase inhibitors, thromboxane receptor antagonists, prostaglandin E2 receptor antagonists, prostacyclin analogs, GP VI receptor antagonists, and thrombin receptor antagonists [3]. Aspirin is a nonsteroidal anti-inflammatory drug that inhibits both inflammation and platelet function, thus making it a commonly used drug for the treatment of cardiovascular disease [2,7]. Aspirin inhibits cyclooxygenase (Cox) in platelets [2,8] and reduces the motility, proliferation, and progression of cancer [2,7,8,9]. Clopidogrel, prasugrel, and ticagrelor (all purinergic P2Y12 receptor inhibitors) are often used as dual therapies in combination with aspirin [10,11]. After absorption through the intestine, clopidogrel and prasugrel can act on platelets after they are activated by cytochrome (Cyp) enzymes in the liver [12,13]. The mechanism is slightly different for ticagrelor, which acts directly on platelets after being absorbed and is then acted on by Cyp [12]. Clopidogrel binds to P2Y12 receptors on platelets to inhibit platelet function and, consequently, inhibit the motility, proliferation, and progression of cancer cells [3,12,14]. Furthermore, experiments in a mouse model confirmed that ticagrelor inhibits platelet function and reduces cancer cell metastasis [14].

Previous studies have shown no increase in noncardiovascular mortality in patients treated with clopidogrel in the CAPRIE and CHARISMA clinical trials [3,15]. Moreover, a recent systematic review and meta-analysis derived from six randomized controlled trials and three cohort studies revealed that the cancer event rate did not differ between patients exposed to prasugrel or clopidogrel when compared to those treated with aspirin or a placebo [3,15]. Various clinical studies and in vivo and in vitro experiments have investigated the relationship between antiplatelet agents and cancer [2,3,7,8,9,10,14,15].

Interestingly, data have emerged to suggest that the risk for solid cancers is increased in patients treated with these medications; however, this remains controversial [16,17,18,19]. Food and Drug Administration reports suggested that high doses of prasugrel are related to an increase in cancer incidence [20]. Clinical data from the TRITON-TIMI 38 clinical trial showed that more new cancer cells were generated in patients receiving prasugrel than in those receiving clopidogrel [3,17]. In the Dual Antiplatelet Therapy trial, cancer mortality was increased in patients who received long-term clopidogrel and prasugrel therapy [16]. In addition, ticagrelor administration was correlated with cancer development in the Prevention of Cardiovascular Events in Patients with Prior Heart Attack Using Ticagrelor Compared to Placebo on a Background of Aspirin-Thrombolysis in Myocardial Infarction 54 (PEGASUS-TIMI) clinical trial [16,21].

Most studies investigating the effects of antiplatelet agents on cancer have been conducted by adding platelets to cancer cell culture experiments and mouse model experiments [7,8,9,14]. This study is conducted to confirm clinical observations that have correlated antiplatelet agents with an increased incidence of solid tumors [16,17,18,20]. Unlike previous reports, the incidence of cancer has increased in patients receiving long-term antiplatelet therapy, so we investigate the direct effects of antiplatelet agents on cancer cells in a platelet-free environment. Since antiplatelet agents are known to be absorbed through the intestines [12], the effect on cancer cells is investigated by directly treating colon cancer cells with antiplatelet agents. 

In this study, we identify the mechanisms involved in cancer cell mobility induced by directly treating antiplatelet agents to cancer cells. These results suggest that SERPINE1 can be used as a valuable marker for predicting cancer incidence and metastasis in patients receiving long-term antiplatelet treatment. 

## 2. Results

### 2.1. Purinergic Antiplatelet Agents Reduce Proliferation While Increasing the Mobility of Colon Cancer Cells

Cancer-related thrombosis is known as an important sign of malignant tumors, and numerous antiplatelet agents have been known to inhibit the proliferation and metastasis of such cancers [1,2,3,14]. Purine P2Y12 inhibitors act to inhibit platelet aggregation by inhibiting adenosine diphosphate P2Y12 receptors [12], and increased cancer mortality has been reported in patients with long-term use of these drugs [11,12,13,14,15]. 

**Figure 1:**
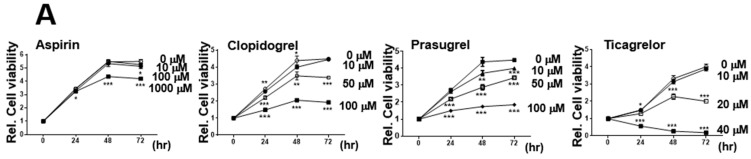

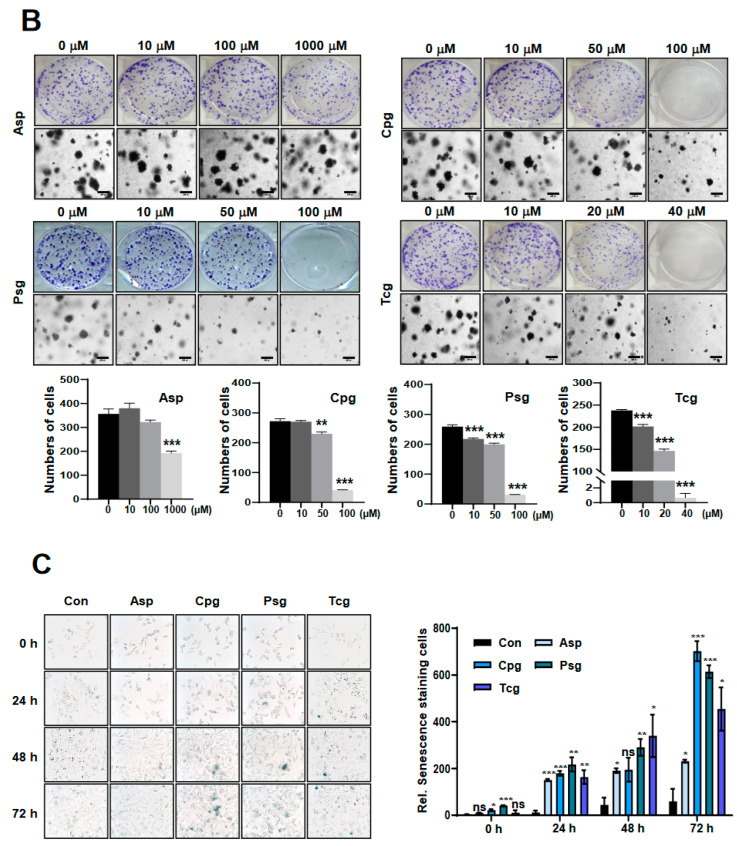
Antiplatelet reagents reduce the proliferation of HCT116 colon cancer cells. (**A**) Cell proliferation was analyzed by MTT in HCT116 cells treated with Asp and 3 antiplatelet agents (clopidogrel, Cpg; prasugrel, Psg; and ticagrelor, Tcg) at the indicated concentrations for 24, 48, and 72 h. (**B**) In the clonogenic assay, HCT116 cells were incubated with Asp or 3 antiplatelet agents at the indicated concentrations for 14 days. Cells were stained with crystal violet solution, and viable colonies were counted (below graph). Columns indicate the numbers of colonies from three independent experiments. HCT116 cells treated with Asp and 3 antiplatelet agents at the indicated concentrations for 14 days were assayed for anchorage-independent growth in soft agar. Representative images were captured with a microscope (magnification ×200). (**C**) SA-β-gal activity in HCT116 cells was confirmed 24, 48, and 72 h after treatment with Asp and 3 antiplatelet agents, respectively, at predetermined concentrations. Representative images were captured with a microscope (magnification ×200). In (**A–C**) data are shown as means ± SD. Statistical significance was determined using ordinary two-way ANOVA; ns, not significant; *, *p* < 0.05; **, *p* < 0.01, and ***, *p* < 0.001; *n* ≥ 3 per group.

In this study, the effect of aspirin and these three purine antiplatelet agents on the proliferation of cancer cells was confirmed by MTT analyses at various concentrations of antiplatelet agents (Figure 1A). With all four antiplatelet agents, cell proliferation decreased as the concentration increased (Figure 1A). As a result of the MTT assay, subsequent experiments were conducted at concentrations of agents that did not affect cell viability: aspirin (Asp) at 1000 µM, clopidogrel (Cpg) at 100 µM, prasugrel (Psg) at 100 µM, and ticagrelor (Tcg) at 20 µM. We performed colony formation assays to determine whether the antiplatelet agents affected the sphere formation of cancer cells (Figure 1B). The reduction in sphere formation with the increase in the concentration of each antiplatelet agent was similar to the patterns observed in the MTT assay described above (Figure 1A,B). It was confirmed whether an increase in apoptosis caused this decrease in cell proliferation, but no significant difference was observed due to treatment with the above agents. Next, the correlation between cell proliferation reduction and cellular senescence was investigated. As a result, more senescence-stained cells appeared as time increased after incubation in transfected cells with pSERPINE1 compared to the control group (Figure 1C). 

In clinical practice, purinergic P2Y12 receptor inhibitors are often used as a dual therapy in combination with aspirin [10,22], so we also treated the cells with aspirin combined with each of the purine antiplatelet agents to compare the results of the group treated with antiplatelet agents alone (Figure S1A). The results showed that cell proliferation was further reduced when cells were co-treated with aspirin compared to the treatment of purinergic P2Y12 receptor inhibitors alone (Figure S1A). 

**Figure 2:**
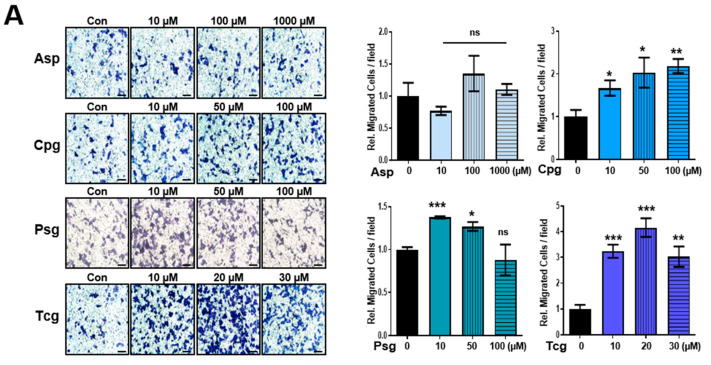

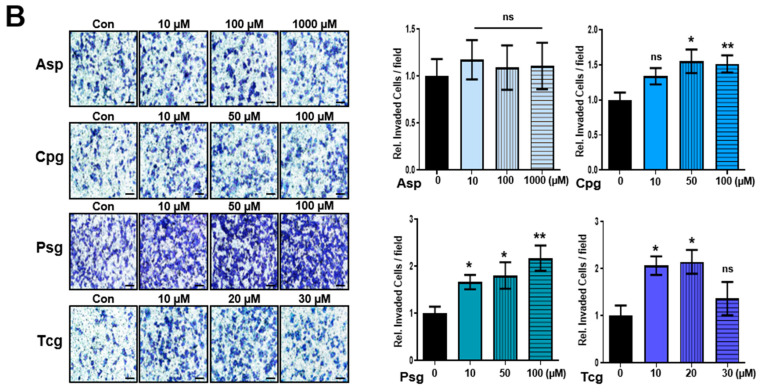
The antiplatelet agents promote HCT116 colon cancer cell migration and invasion ability. (**A**,**B**) Cell migration and invasion assays were performed using a Boyden chamber assay after treatment with aspirin (Asp) or antiplatelet agents (clopidogrel, Cpg; prasugrel, Psg; and ticagrelor, Tcg;) at the indicated concentrations in HCT116 colon cancer cells. Cells were incubated for 24 h in membranes precoated with collagen for the migration assay, with Matrigel for the invasion assay. Scale bar is 100 µm. In (**A**,**B**), data are shown as means ± SD. Statistical significance was determined using ordinary two-way ANOVA; ns, not significant; *, *p* < 0.05, **, *p* < 0.01, and ***, *p* < 0.001; *n* ≥ 3 per group.

Previous studies have shown decreased cancer cell mobility after antiplatelet treatment in a platelet-containing environment [3,7,8,9]. On the other hand, this study aimed to determine the effect of antiplatelet agents on cancer cell motility in a platelet-free environment to compare the effects of patients receiving long-term antiplatelet therapy. Aspirin or each of three purinergic antiplatelet agents was administered to cancer cells at the same concentrations as in the above experiment, respectively, and the migration and invasion of cancer cells were examined by Boyden chamber assay (Figure 2A,B). Aspirin did not affect the migration and invasion of cancer cells, but the other three purinergic antiplatelet drugs showed significant increases in the migration and invasion of cancer cells (Figure 2A,B). Cancer cells treated with low concentrations of ticagrelor or prasugrel showed higher cell migration (migration and invasion) (Figure 2A,B). In addition, since these purinergic antiplatelet agents are often used as a dual therapy in combination with aspirin in the clinic [10], the co-treatment effects with these agents and aspirin were compared for cell invasion. Compared to treatment with purinergic P2Y12 receptor inhibitors alone, co-treatment with aspirin did not affect, or rather reduced, the invasion of cancer cells (Figure S1B).

### 2.2. Gene Expression Profile Analysis to Identify Genes Associated with Cancer Cell Mobility 

**Figure 3:**
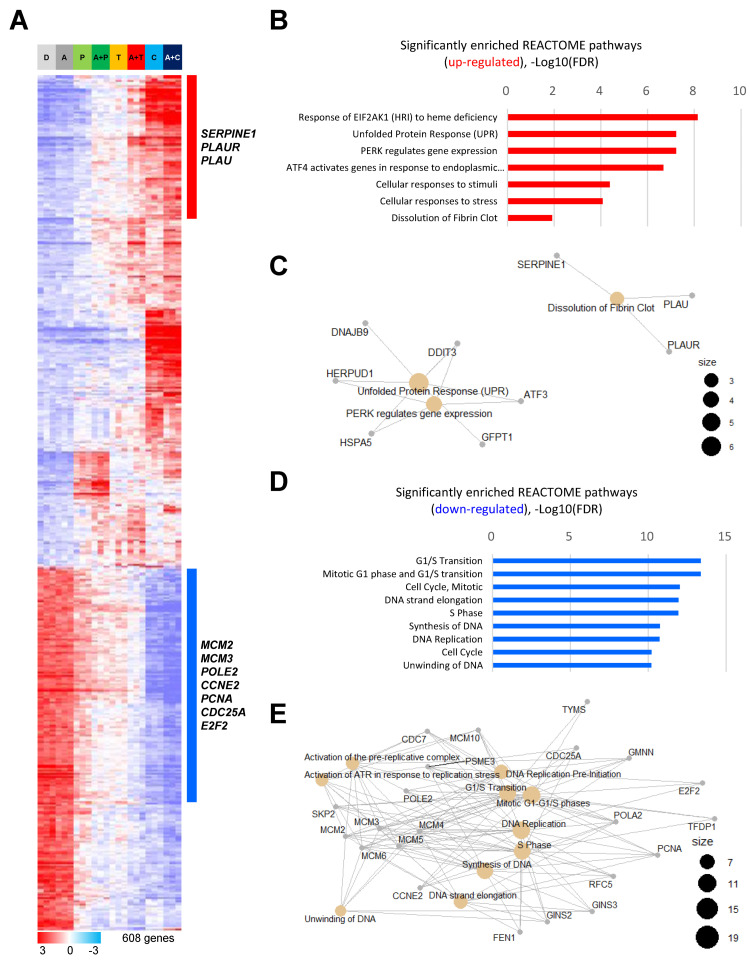
Microarray gene expression profiling and biological pathways related to antiplatelet agent treatments. (**A**) A transcriptional pattern of 608 differentially expressed genes. Student’s *t*-tests were applied to gene expression profile data from two groups of samples (DMSO and aspirin vs. three antiplatelet agents), and genes were considered statistically significant (*p* < 0.05 and 1.5-fold change). Positive (red) and negative (blue) expression correlations are shown. D, DMSO; A, aspirin; P, prasugrel; T, ticagrelor; C, clopidogrel; A+P, aspirin and prasugrel; A+T, aspirin and ticagrelor; A+C, aspirin and clopidogrel. (**B**) A graph analyzing gene ontology (GO) based on positively expressed genes. (**C**) Gene concept network analysis with reactome based on gene ontology (GO) analysis. (**D**) A graph analyzing gene ontology (GO) based on negatively expressed genes. (**E**) Gene concept network analysis with reactome based on GO analysis.

Since the causes of increased mobility of cancer cells by treatment with antiplatelet agents are unknown, we analyzed gene expression profile data using drug-treated colon cancer cell lines (HCT116). A clustering analysis was performed using 608 genes with differences in gene expression between the DMSO, aspirin group and the three antiplatelet agent groups (prasugrel, ticagrelor, and clopidogrel). As a result, we confirmed that the gene expression of colon cancer cell lines was dramatically changed by clopidogrel treatment (Figure 3A).

We performed a gene ontology (GO) analysis using reactome to identify biological pathways that changed after antiplatelet treatment. We observed changes in various biological pathways and identified increased expression levels of genes related to the dissolution of fibrin clots (PLAU, PLAUR, and SERPINE1) directly associated with antiplatelet treatment (Figure 3B,C). On the other hand, it was confirmed that DNA proliferation- and replication-related genes (MCM2, MCM3, POLE2, CCNE2, PCNA, CDC25A, and E2F2) were decreased by antiplatelet treatment (Figure 3D,E). 

In addition, to obtain genes associated with increased apoptosis and cell motility following antiplatelet treatment, we identified 516 genes that are commonly involved in the apoptotic process and cell motility from MSigDB (http://gsea-msigdb.org, accessed on 20 July 2022). We also selected 116 genes that showed differential expression after three antiplatelet treatments. Eight genes (ARHGEF2, BMP4, BTG1, DDIT4, FOXC1, SH3KBP1, TNFRSF10B, and SERPINE1) were commonly included in both cases (Figure S2A–C). The expression patterns of the eight genes were confirmed by the clustering analysis (Figure S3A). Considering the statistical significance and molecular characteristics of the eight genes, we selected SERPINE1 and confirmed that SERPINE1 was associated with decreased cell proliferation and increased cell motility in colon cancer cells (Figure S3B).

### 2.3. Cancer Cells Treated with Purinergic Antiplatelet Agents Induce the Expression of SERPINE1

**Figure 4:**
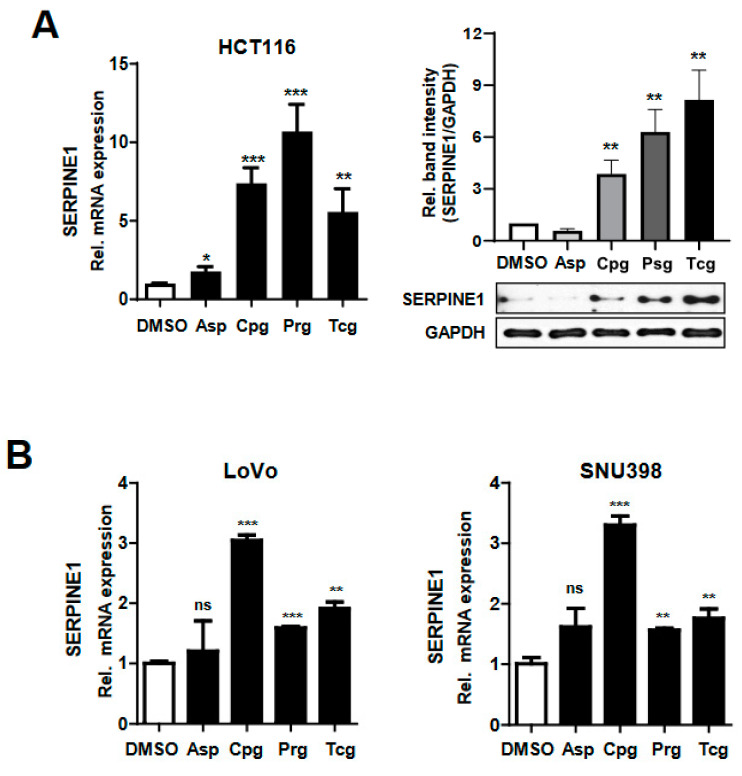

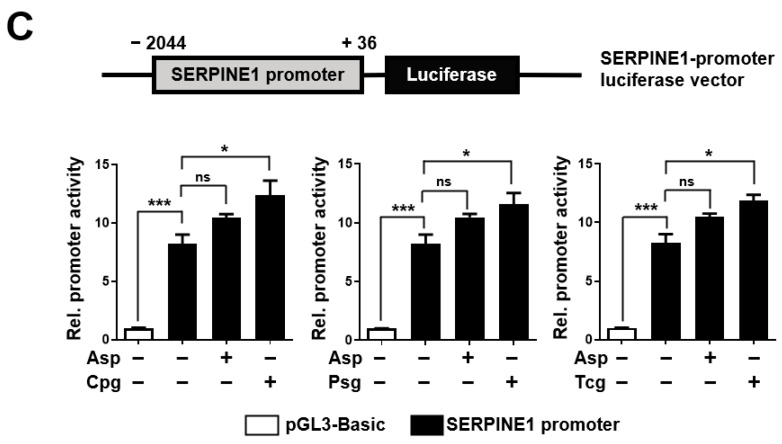
SERPINE1 expression and promoter activity increase in HCT116 colon cancer cells treated with antiplatelet agents. (**A**) HCT116 cells were treated for 24 h with DMSO (as a control), Asp (1000 μM), Cpg (100 μM), Psg (100 μM), or Tcg (20 μM). SERPINE mRNA and protein expression was assessed by qRT-PCR and Western blotting. SERPINE1 protein band intensity was quantified by density using ImageJ software. The bar graph shows the relative expression density of SERPINE1 normalized to GAPDH levels. (**B**) LoVo and SNU398 cells were treated for 24 h with DMSO, Asp, Cpg, Psg, or Tcg. SERPINE mRNA and protein expression was assessed by qRT-PCR. (**C**) Upper panel shows a schematic representation of the SERPINE1 promoter region with the luciferase gene in the pGL3 vector. The antiplatelet agents were applied to HCT116 cells containing the SERPINE1 promoter vector and measured using a luciferase assay. Statistical significances for all data were determined using ordinary two-way ANOVA; ns, not significant; *, *p* < 0.05; **, *p* < 0.01, and ***, *p* < 0.001; *n* = 3 per group; compared to the pGL3-based construct.

SERPINE1 expression, assessed by microarray experiments, was confirmed at the mRNA and protein levels by qRT-PCR and Western blotting (Figure 4A). There was no difference in the mRNA and protein levels between the control group and the aspirin-treated group, but *SERPINE1* expression was significantly increased when the cells were treated with each purinergic antiplatelet agent (Figure 4A). In order to confirm that the expression of SERPINE1 was specific to the cell line, other colorectal cancer cells (LoVo cell line) and the liver cancer cell line (SNU398 cell line) were additionally used (Figure 4B). As a result, the expressions of SERPINE1 were significantly increased in the other two types of cancer cell lines when treated with each of the three purinergic antiplatelet agents compared to the control and aspirin-treated cell lines (Figure 4B). 

Next, the SERPINE1-promoter luciferase vector was constructed, and it was investigated whether the expression of SERPINE1 was controlled by these agents using a luciferase analysis (Figure 4C). When luciferase activity was examined 24 hours after each antiplatelet treatment, a significant increase was observed in cancer cells transfected with the SERPINE1 promoter luciferase vector compared to those transfected with the control vector (pGL3-based vector) (Figure 4C). These results indicate that SERPINE1 expression was induced when each of the three antiplatelet agents was directly applied to the cancer cells.

**Figure 5:**
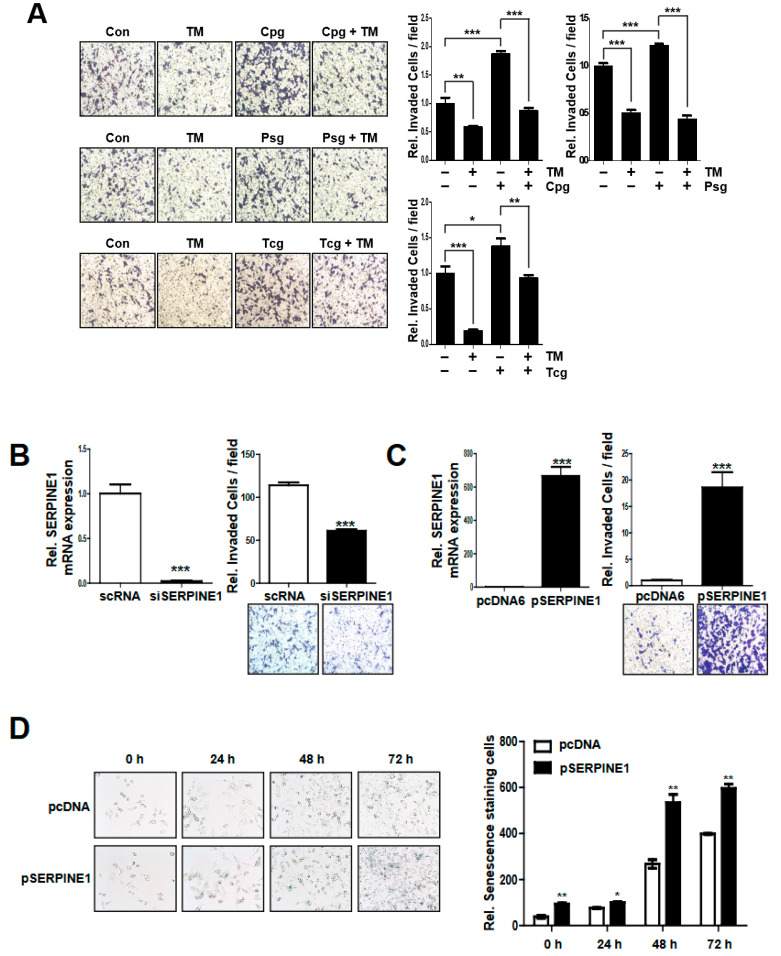
Inhibition of SERPINE1 decreases the invasion ability of HCT116 colon cancer cells. (**A**) TM5275, a small molecule inhibitor of SERPINE1, inhibits the migration of HCT116 colon cancer cells. Cell invasion was assessed using a Boyden chamber assay after treatment with TM5275 and antiplatelet agents at the indicated concentrations in HCT116 colon cancer cells. Cells were incubated for 24 h in membranes pre-coated with Matrigel for the invasion assay. (**B**) SERPINE1 expression in HCT116 cells after transfection with siSERPINE1 or scRNA. Cell invasion ability was measured using Boyden chamber assays at 24 h post-transfection with siSERPINE1. (**C**) SERPINE1 expression in HCT116 cells after transfection with SERPINE1 overexpression vector (pSERPINE1) or pcDNA6. Cell invasion ability was measured using Boyden chamber assays at 24 h post-transfection with pSERPINE1. (**D**) SERPINE1 expression in HCT116 cells after transfection with SERPINE1 overexpression vector (pSERPINE1) or pcDNA6. Senescence staining was confirmed at 24, 48, and 72 h after transfection. Representative images were captured with a microscope (magnification ×200). Statistical significances for these data were determined using ordinary two-way ANOVA and Student’s *t*-tests; *, *p* < 0.05, **, *p* < 0.01, and ***, *p* < 0.001; *n* ≥ 3 per group.

To investigate the correlation between cancer cell mobility and SERPINE1 expression level, the effect of the SERPINE1 expression level on cell invasion was investigated. TM5275 (Figure S4A), known as an inhibitor of SERPINE1 [23], was used, and its effect on the cell mobility of cancer cells was assessed. To determine the optimal concentration of TM5275, cell viability was investigated by MTT assay after treatment with concentrations of 0, 10, 50, and 100 μM (Figure S4B). A dose of 50 μM TM5275, which had a minimal effect on cell survival, was applied to subsequent experiments. The effects on cancer cell invasion were investigated after using each antiplatelet agent, TM5275, or two agents simultaneously (Figure 5A). Compared to the control, the treatment with TM5275 showed a significant decrease in cell invasion (Figure 5A). In addition, the cell invasiveness increased by treatment with each antiplatelet agent was reduced to control levels when co-treated with TM5275 and each antiplatelet agent (Figure 5A). 

In addition, cell mobility was confirmed by the suppression of SERPINE1 expression using siRNA. When three different siSERPINE1 (#1 to #3) variants were used to check the suppression of SERPINE1 expression, #3 siSERPINE1 showed the most significant suppression (Figure S4C,D) and was used in the subsequent experiment. When this #3 siSERPINE1 was used to suppress the expression of SERPINE1, a decrease in cell invasion was confirmed (Figure 5B). Conversely, a SERPINE1 overexpression plasmid was constructed using the pcDNA6/V5-HisB expression vector (Figure 5C). When the SERPINE1 overexpressing vector was transfected into cancer cell lines, the invasiveness of the cancer cells was significantly increased (Figure 5C). 

In this study, it was confirmed that cancer cell lines treated with purinergic antiplatelet agents increased cell mobility but decreased cell proliferation. To confirm whether this reduction in cell proliferation was associated with SERPINE1 expression, senescence staining was performed on cancer cell lines transfected with pSERPINE1. As a result, more senescence-stained cells appeared as time increased after incubation in transfected cells with pSERPINE1 compared to the control group (Figure 5D). 

### 2.4. Overexpressed SERPINE1 Treated with Purinergic Antiplatelet Agents Increases Cell Mobility by Inducing MMP1 Expression

In order to understand the mechanisms related to SERPINE1 and cell mobility, we focused on the epithelial–mesenchymal transition (EMT) pathway of cancer cell mobility [24]. Many studies have shown that E-cad decreases and N-cad, Snail, Slug, Twist, and Zeb1 increase during metastasis [24,25]. Therefore, the mRNA expressions of EMT factors (E-cad, N-cad, Snail, Slug, Twist, Zeb1, and MMPs) were analyzed using the gene expression profile data of TCGA colon cancer (Figure S5A). In the analysis using TCGA colon cancer data, SERPINE1 and various EMT factors showed a high correlation (Figure S5A). The expressions of 12 EMT factors among them were investigated in control and cancer cells treated with aspirin or purinergic antiplatelet agents. MMP1 expression showed a significant increase in all cancer cells treated with each antiplatelet agent (Figure S5B). A DAVID analysis was performed by selecting genes positively correlated with the expression patterns of SERPINE1 and MMP1 from the TCGA colon cancer cohort. We identified several biological features, such as chemotaxis and the positive regulation of cell migration, indicating increased cell motility (Figure S6A–C).

**Figure 6:**
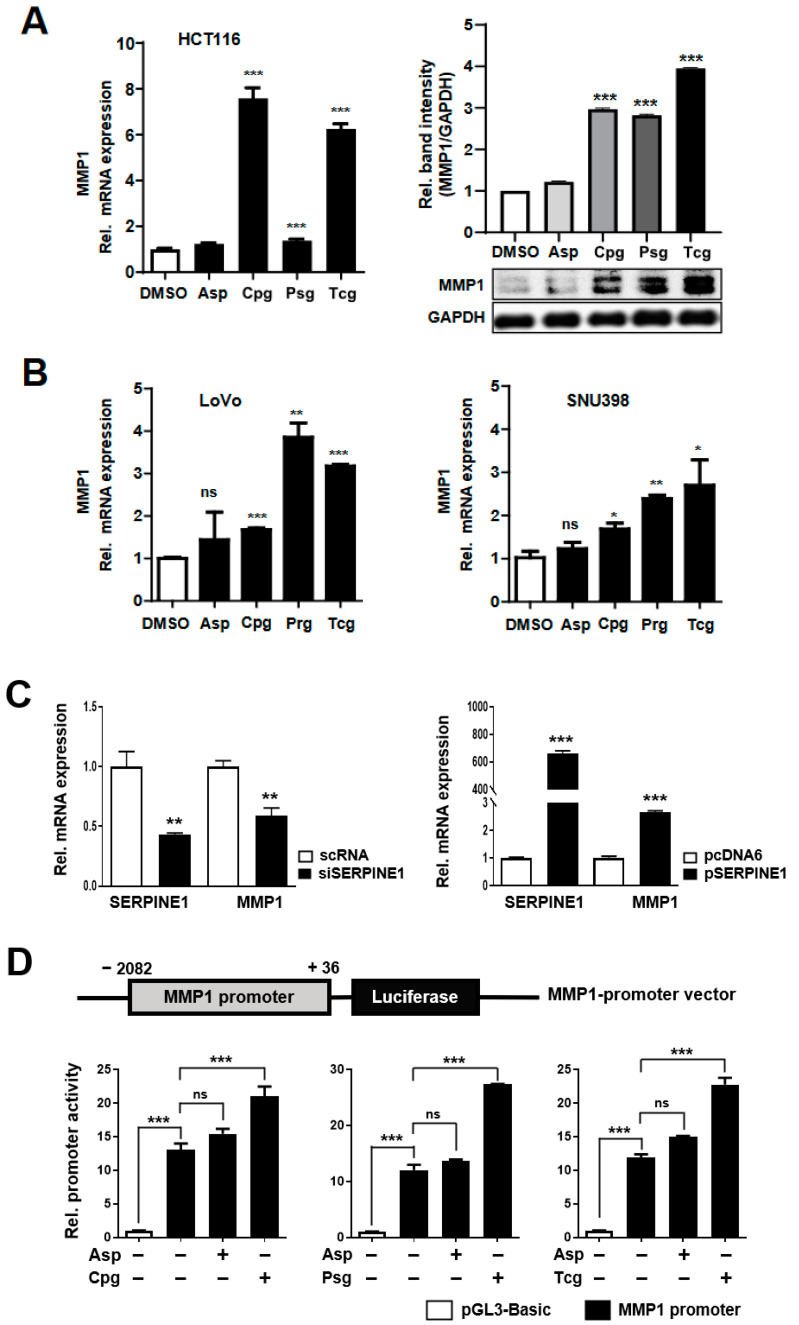
MMP1 expression and promoter activity increases in HCT116 colon cancer cells treated with the antiplatelet agents. (**A**) HCT116 cells were treated for 24 h with DMSO (as a control), Asp (1000 μM), Cpg (100 μM), Psg (100 μM), or Tcg (20 μM). MMP1 mRNA and protein expressions were assessed by qRT-PCR and Western blotting. MMP1 protein band intensity was quantified by density using ImageJ software. The bar graph shows the relative expression density of MMP1 normalized to GAPDH levels. (**B**) LoVo and SNU398 cells were treated for 24 h with DMSO, Asp, Cpg, Psg, or Tcg. MMP1 mRNA and protein expressions were assessed by qRT-PCR. (**C**) HCT116 cells were transfected with SERPINE1 siRNA (siSERPINE1) and SERPINE1 overexpression vector (pSERPINE1) for 24 h. qRT-PCR was performed to detect the mRNA levels of SERPINE1 and MMP1. (**D**) Upper panel shows a schematic representation of the MMP1 promoter region with the luciferase gene in the pGL3 vector. The antiplatelet agents were applied to HCT116 cells containing the SERPINE1 promoter vector and measured using a luciferase assay. In (A-D), data are shown as means ± SD. Statistical significance was determined using ordinary two-way ANOVA and Student’s *t*-tests; ns, not significant; *, *p* < 0.05; **, *p* < 0.01 and ***, *p* < 0.001; *n* ≥ 3 per group.

Therefore, the induction of MMP1 in cancer cells treated with antiplatelet agents was investigated at the mRNA and protein levels by qRT-PCR and Western blotting. (Figure 6A). As in the results of SERPINE1, there was no difference in the mRNA and protein expressions of MMP1 between the control and the aspirin-treated groups. Interestingly, when each purinergic antiplatelet agent was treated, the expression of MMP1 showed a significant increase similar to that of SERPINE1 (Figure 6A). This increase in MMP1 was similarly shown when the LoVo and SNU398 cell lines were used, as in SERPINE1 (Figure 6B). In addition, the expression of MMP1 was decreased in the cells transfected with siSERPINE, and conversely, the expression of MMP1 was increased by the overexpression of SERPINE1 (Figure 6C).

An MMP1 promoter luciferase vector was also constructed to examine the expression of MMP1 after treatment with antiplatelet agents (Figure 6D). Cells transformed with the MMP1 promoter vector showed a significant increase in luciferase activity compared to those in the control (pGL3-based vector) (Figure 6D). 

These results suggest that the direct treatment of antiplatelet agents to cancer cells induced the expressions of SERPINE1 and MMP1 to promote cell mobility.

### 2.5. Secretory SERPINE1 Protein, Which Can Be Used as a Biomarker for Antiplatelet Agents Therapy 

**Figure 7:**
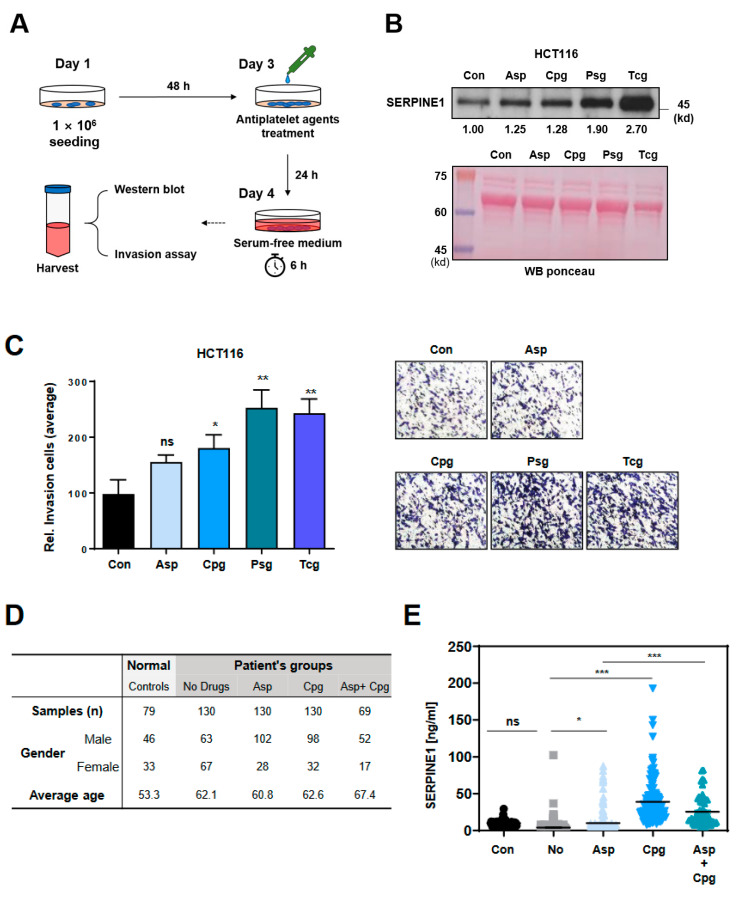
SERPINE1 secreted by antiplatelet agents increases invasion ability. (**A**) Schematic showing the workflow for harvesting conditioned medium (CM) from cells treated with antiplatelet agents. (**B**) After HCT116 colon cancer cells were treated with Asp (1000 μM), Cpg (100 μM), Psg (100 μM), and Tcg (20 μM) for 24 h, CM was collected and SERPINE1 expression was confirmed by Western blotting. Ponce staining was presented as a loading control. (**C**) The cell invasion assay was cultured using a Boyden chamber for 48 h with HCT116 colon cancer cells and CM of cells treated with aspirin (Asp) or antiplatelet agents (Cpg, Psg, and Tcg). (**D**,**E**) SERPINE1 concentrations were measured in plasma samples from control (*n* = 79), non-drug treated patients (*n* = 130), Asp-treated patients (*n* = 130), Cpg-treated patients (*n* = 130), and Asp+Cpg-treated patients (*n* = 69) via ELISA assay. Statistical significance was determined using ordinary two-way ANOVA and Student’s *t*-tests; ns, not significant; *, *p* < 0.05, **, *p* < 0.01, and ***, *p* < 0.001; *n* ≥ 3 per group.

According to previous reports, SERPINE1 is known as a secreted protein [26]. In particular, it has been reported that the secretion of SERPINE1 is increased in cancer cells, which affects cell mobility [27,28]. Therefore, it was examined whether SERPINE1 was secreted from HCT116 cells treated with each antiplatelet agent. The cells were treated with each antiplatelet agent for 24 hours and then the medium was replaced with conditioned medium (CM), and the CM was recovered after 6 hours (Figure 7A). The increase in SERPINE1 protein in the CM was confirmed by Western blotting (Figure 7B). As a result, the antiplatelet agent-treated groups showed an increase in secreted SERPINE1 compared to the control group (Figure 7B). It was also investigated whether secreted SERPINE1 affected cell invasion by treating CM containing SERPINE1 secreted from cells (Figure 7C). There was no significant difference between the control and the aspirin-treated groups, but cell invasion was significantly increased in the purine-antiplatelet-treated group (Figure 7C). 

Next, it was confirmed whether the secretion of SERPINE1 was detected in a patient who received the antiplatelet drug through the patient’s liquid biopsy (Figure 7D,E). An ELISA assay was performed to determine the concentration of SERPINE1 in a total of 538 serum samples from a normal control group (79), a non-drug patient group (130), Asp-(130) or Cpg-(130) single-treated groups, and an Asp and Cpg combined treatment group (69) (Figure 7D,E). All the groups included patients who had taken the agents for at least 6 months. Patients taking antiplatelet agents other than aspirin or clopidogrel were excluded from this analysis because samples were not available. In the case of combined administration, only aspirin and clopidogrel samples were secured, and the experiment was carried out. 

There was no significant difference between the normal control group and the non-drug patient group (patients who did not take drugs), which considered no secretion of SERPINE as a characteristic of the patients (Figure 7E). In contrast, the concentrations of SERPINE1 were found to be significantly higher in patients who took aspirin and clopidogrel alone or in combination (Figure 7E). Therefore, these results show that treatment with antiplatelet agents was associated with the secretion of SERPINE1. However, the patients recruited in this study had intra-group or inter-group differences in the treatment period of antiplatelet agents, so this result was somewhat limited.

## 3. Discussion

Activated platelets help cancer cells attach to the blood vessel walls and are involved in the metastasis of cancer cells [1]. Thus, various antiplatelet agents, including COX inhibitors and P2Y12 receptor inhibitors, have been reported to inhibit cancer cell metastasis [3,7,8,9,10]. Conversely, it has been reported that mortality in cancer patients receiving long-term treatment with antiplatelet agents has increased [12]. Therefore, this study investigated the effect of antiplatelet agents applied directly to cancer cells in a platelet-free environment. 

In this study, it was confirmed that antiplatelet agents reduced the proliferation of colon cancer cells, similar to the results of previous studies [3,7,8,9]. In contrast, treatment with P2Y12 receptor inhibitors actually increased the mobility of cancer cells. These results were inconsistent with other results for P2Y12 receptor inhibitors [9]. This difference could be seen as the effect of the direct treatment on cancer cells in a platelet-free environment in this study, unlike previous studies where platelets have been present.

To investigate the mechanisms involved in exhibiting these characteristics, SERPINE1 was identified as a key gene by analyzing the whole gene expression profile. Increased expression of SERPINE1 was confirmed by the direct treatment of colon cancer cells with a purine-based antiplatelet agent, and this overexpression suggested a correlation with metastasis as the cell mobility of cancer cells increased. Similar to the results of this study, it has been reported that the overexpression of SERPINE1 in several cancer cell lines increases cell mobility and decreases proliferation [29,30]. According to recent studies, the expression of SERPINE1 has been associated with progression and poor prognosis in colorectal cancer [31,32], suggesting that SERPINE1 can be used as a prognostic marker [33,34,35,36].

The protein encoded by SERPINE1 is called plasminogen activator inhibitor-1 (PAI-1), also known as endothelial plasminogen activator inhibitor, and elevated PAI-1 is known as a risk factor for thrombosis and atherosclerosis [23,30,37]. On the other hand, in this study, the expressions of uPAR and uPA were increased with the increase in SERPINE1 in cancer cells treated with purine antiplatelet agents. These results suggested that treatment with antiplatelet agents may also be involved in the metastasis of cancer cells, which conflicts with many studies [5], showing that activated platelets are associated with cancer cell survival and metastasis [23,38]. Thus, the results of this study may support the hypothesis that patients with long-term antiplatelet therapy have increased cancer mortality [39,40]. Therefore, on the basis of these results, the possibility of new cancer development and cancer metastasis in patients undergoing long-term treatment with antiplatelet agents can be investigated to confirm the level of the secretory protein PAI-1 [28], as well as to explore ways to eliminate the side effects of these agents. MMP1 is included as an EMT-related marker gene in a public database (MsigDB). Among the candidate genes, MMP1 positively correlated with SERPINE1 in the TCGA colorectal cancer cohort (R = 0.51). In addition, through a gene ontology analysis of common genes showing a positive correlation between SERPINE1 and MMP1, it was suggested that the two genes were indirectly related to EMT. 

Although this study, mainly including in vitro experiments, may have limitations from a clinical point of view, especially for patients who have been treated with antiplatelet agents for a long time and have cancer at the same time, they may be administered considering the characteristics of antiplatelet agents. The need to administer agents that inhibit SERPINE1 should also be considered as an alternative to reduce the risk of cancer cell metastasis.

## 4. Materials and Methods

### 4.1. Cell Culture and Reagents

The human colon cancer cell lines HCT116 and LoVo and the human liver cancer cell line SNU398 were purchased from the American Type Culture Collection (ATCC; Manassas, VA, USA) and cultured in RPMI medium (Capricorn Scientific, Ebsdorfergrund, Germany) containing 10% FBS (Capricorn Scientific, Ebsdorfergrund, Germany) and penicillin/streptomycin (Capricorn Scientific, Ebsdorfergrund, Germany). The cells were maintained at 37ºC in a humidified incubator with an atmosphere of 5% CO_2_. Antiplatelet agents, aspirin (Cat #A3160), clopidogrel (Cat #C0614), prasugrel (Cat #SML0331), and ticagrelor (Cat #CDS023238) were purchased from Sigma-Aldrich (St.Louis, MO, USA). TM5275 sodium salt, which is known to inhibit SERPINE1, was purchased from Tocris Bioscience (Cat #5769, Bristol, Avon, UK).

### 4.2. MTT Assay, Clonogenic Assay, and Soft Agar Assay

Cell proliferation rates were determined with a 3-(4, 5-dimethylthiazole2-yl)-2, 5-diphenyl tetrazolium bromide (MTT) assay (Cat #M2128, Sigma-Aldrich, St. Louis, MO, USA), as described previously [41]. Clonogenic assays were performed to observe cancer cell proliferation in the adherent state, as described previously [41]. A soft agar assay was performed to confirm cancer cell proliferation in the floating state. The bottom agar was a cell-free layer. This was plated onto a 6-well culture plate with media containing 0.7% novel agar (Cat #214230, Becton, Dickinson and Company, Franklin Lakes, NJ, USA) and 10% FBS and was left to solidify at room temperature on a clean bench. The top layer of agar contained cells. This media contained 0.37% novel agar, 10% FBS, and antiplatelet agents and was plated on top of the bottom agar and left to solidify at room temperature on a clean bench. Media containing antiplatelet agents was added to the top agar, and 500 µL of media was added every 3 days. The plates were incubated at 37 °C in a humidified incubator (5% CO_2_) for 2 weeks.

### 4.3. Senescence Staining

Before starting the staining procedure, 1 × 10^5^ cells were seeded in 6-well plates. At 24 h after seeding, each plate was treated at concentrations of 1000 µM for aspirin, 100 µM for clopidogrel, 100 µM for prasugrel, and 20 µM for ticagrelor for 0, 24, 48, and 72 h. Cell senescence was detected using a Senescence β-Galactosidase cell staining kit (Cat #9860, Cell Signaling Technology, Danvers, MA, USA) according to the manufacturer’s instructions. Briefly, the cell media was removed, washed with PBS, and then fixed with a fixative solution at room temperature for 10–15 minutes. Then, it was washed twice with PBS, β-Galactosidase staining solution prepared in advance was added, and it was incubated overnight at 37 °C without CO_2_. Blue color was observed under a microscope at × 200 magnification.

### 4.4. Invasion and Migration Assay

A standard 48-well chemotaxis chamber (Cat #AP48, Neuro Probe Inc., Gaithersburg, MD, USA) was used to confirm cell invasion and migration. Membranes (Cat #PFB8, Neuro Probe Inc., Gaithersburg, MD, USA) with an 8 µm pore size were precoated with Matrigel (Cat #354234, Corning, Costar, NY, USA) and collagen (Cat #C7661, Sigma-Aldrich, St.Louis, MO, USA) to confirm invasion and migration. In the bottom chamber, 30 µL of media containing 1% FBS was added as a chemoattractant. Cells were suspended in FBS-free media and seeded at 5 × 10^4^ cells/56 µL in the upper wells with antiplatelet agents. After 24 h, the membranes were stained with Diff Quik reagent (Cat #38721, Sysmex Co., Kobe, Japan). After attaching the membranes to glass plates, the cells that passed through the membranes were observed through an Axiovert 40 CFL microscope (Carl Zeiss, Oberkochen, Germany).

### 4.5. Quantitative Real-Time Polymerase Chain Reaction (qRT-PCR)

The total RNA was subsequently isolated from cells using RNAiso Plus (Cat #9109, Takara, Otsu, Japan), as described previously [41]. Subsequently, cDNA was synthesized using a PrimeScript^TM^ RT reagent kit (Cat #RR036A, Takara, Otsu, Japan). qRT-PCR was performed using SYBR Premix Ex Taq (Cat #RR420A, Takara, Otsu, Japan) and a CFX96^TM^ Optical Reaction Module (Cat #184-5096, BioRad, Hercules, CA, USA). Genes were normalized against the control GAPDH. The primers for qRT-PCR are listed online in Supplementary Table S1.

### 4.6. Western Blot Analysis and Luciferase Assay

Cells were washed with PBS, dissolved in 200 µL of radioimmunoprecipitation assay (RIPA) buffer containing protease inhibitor (Cat #04693116001, Roche, Mannheim, Germany), and sonicated. Protein levels were measured using a Pierce^TM^ BCA Protein Assay kit (Cat #23227IL, Thermo Fisher Scientific, Waltham, MA, USA). Primary antibodies against GAPDH (Cat #AbC-1001, AbClon, Seoul, Korea), SERPINE1 (Cat #Ab66705, Abcam, Cambridge, MA, USA), MMP1 (Cat #sc137044, Santa Cruz Biotechnology, Santa Cruz, CA, USA), p38 (Cat #9212S, Cell Signaling Technology, Danvers, MA, USA), and P-p38 (Cat #9211S, Cell Signaling Technology, Danvers, MA, USA) were used.

Cells were seeded in 12-well culture plates, and promoter plasmids containing the MMP1 and SERPINE1 promoters were transfected. Transfected cells were treated with four antiplatelet agents: aspirin (1000 µM), clopidogrel (100 µM), prasugrel (100 µM), and ticagrelor (20 µM). Measurements were performed using a luciferase system. Luciferase activity was measured using a Dual-Luciferase Reporter Assay System (Cat #E1960, Promega, Madison, WI, USA), as described previously [41]. 

### 4.7. Small Interfering RNA, Overexpression Plasmids, Promoter Plasmids, and Transfection 

Small interfering RNA (siRNA) for the human SERPINE1 gene (hs.Ri.SERPINE1.13) was purchased from Integrated DNA Technologies (Coralville, IA, USA). To construct pSERPINE1 (SERPINE1 overexpression vector), the coding sequence (CDS) of SERPINE1 was inserted into the *Hin*dIII/*Xho*I restriction enzyme sites of the pcDNA6/V5-HisB mammalian expression vector (Cat #V220+01, Life Technologies, Carlsbad, CA, USA). The primers used in the plasmid constructs of this study are listed online in Supplementary Table S2. Promoter plasmids containing SERPINE1 and MMP1 were constructed using the pGL3-basic vector (Promega, Madison, WI, USA). Polymerase chain reaction (PCR) was performed using a GeneAmp PCR System 9700 (Applied Biosystems, Foster City, CA, USA).

### 4.8. Conditioned Medium (CM) Preparation, Concentration, and ELISA Assay

HCT116 cells were seeded in a 100 mm plate by 1 × 10^6^ cells and, after 48 h when the cells grew 80%, aspirin (1000 µM) and 3 antiplatelet agents (clopidogrel (100 µM), prasugrel (100 µM), and ticagrelor (20 µM)) were treated according to the prescribed concentration for 24 h. The cell medium was removed and treated with fresh medium without FBS for 6 h. The medium of each plate was harvested and centrifuged for 10 min at 1000 rpm and 4 °C, and cell debris was removed. The collected CM was concentrated with a Vivaspin Column (Cat #28932359, Cytiva, Marlborough, MA, USA).

A Human SERPINE1/PAI-1 DuoSet ELISA kit (Cat #DY1786, R&D systems, Minneapolis, MN, USA) was used according to the manufacturer’s instructions to determine the concentration of SERPINE1 in the human serum. The optical density of each well was determined using a microplate reader set to 450 nm. Then, the 540 nm reading was subtracted from the reading at 450 nm. This subtraction corrected for optical defects in the plate.

### 4.9. Microarray Gene Expression Profiling

Microarray experiments were performed to identify the target genes of the antiplatelet agents. HCT116 cells were treated with various combinations of antiplatelet agents (e.g., aspirin (1000 µM), clopidogrel (100 µM), prasugrel (100 µM), and ticagrelor (20 µM)) for 24 h and then tested according to the manufacturer’s instructions (Illumina, Inc., San Diego, CA, USA). The gene expression data were normalized using quantile normalization in an R 3.6.1 language environment (http://www.r-project.org, accessed on 1 March 2022). Measured gene expression values were log2-transformed and median-centered across genes and samples. Gene expression datasets are available in the National Center for Biotechnology Information (NCBI) Gene Expression Omnibus (GEO) public database under data series accession number GSE153127. 

### 4.10. Statistical Analysis

Before clustering, we selected 608 genes with expression levels that exhibited at least a two-fold difference relative to the median value in greater than 10% of the samples. Using a gene expression data matrix consisting of a gene feature and its correlated genes, we performed a hierarchical clustering analysis with the centered correlation coefficient as the measure of similarity and used the centroid-linkage-clustering method. The mRNA expression (RNA-Seq) data from the Cancer Genome Atlas database were obtained from the cBioPortal website (http://www.cbioportal.org, accessed on 20 July 2022). We downloaded the mRNA quantification data (generated by RSEM software), to which log2 transformation and quantile normalization were applied. To explore the relationship between the genes in the signature, we performed Database for Annotation, Visualization, and Integrated Discovery (DAVID) and reactome analyses (http://reactome.org, accessed on 20 July 2022). In this study, the statistical analyses were performed with Student’s *t*-test, where * *p* < 0.05, ** *p* < 0.01, and *** *p* < 0.001. 

## 5. Conclusions

It was confirmed that purinergic antiplatelet agents can be involved in cancer metastasis by inducing the expression of MMP1, which is involved in cell motility, by increasing the secretion of SERPINE1 in cancer cells while decreasing cancer cell proliferation. Therefore, by confirming the expression of SERPINE1 in patients receiving long-term antiplatelet therapy, it suggests that it can be used as a new target gene to prevent the onset and metastasis of cancer.

## Data Availability

All data generated during this study are included in this published article, and the datasets described in this study can be acquired from the corresponding author upon request.

## Supplementary Materials

The following supporting information can be downloaded at: https://www.mdpi.com/article/10.3390/ijms23179596/s1.
