# Supplementary material for: Secretory SERPINE1 Expression Is Increased by Antiplatelet Therapy, Inducing MMP1 Expression and Increasing Colon Cancer Metastasis
Source: Int J Mol Sci. 2022 Aug 24;23(17):9596. doi: 10.3390/ijms23179596 (PMC9455756; doi:10.3390/ijms23179596)
Supplement: Supplementary file 1 [file ijms-23-09596-s001.zip › ijms-1861048-SI.pdf]

Supplementary Table S1. qRT-PCR primer set

Primer name	Primer sequence	length	Product size
β-Actin F	5’-CCCTGGAGAAGAGCTACGAG-3’	20 mer	132 bp
β-Actin R	5’-AGGTAGTTTCGTGGATGCCA-3’	18 mer	
MMP1 F	5’-TTTGGCTTCCCTAGAACTGTG-3’	21 mer	146 bp
MMP1 R	5’-GCTATCATTTTGGGATAACCTGG-3’	23 mer	
MMP2 F	5’-AATGCCATCCCCGATAACC-3’	19 mer	123 bp
MMP2 R	5’-TCCAAACTTCACGCTCTTCAG-3’	21 mer	
MMP7 F	5’-TCACTTCGATGAGGATGAACG-3’	21 mer	105 bp
MMP7 R	5’-GAATGTCCCATACCCAAAGAATG-3’	23 mer	
MMP9 F	5’-GGAGGTTCGACGTGAAGG-3’	18 mer	130 bp
MMP9 R	5’-TCCTGGCAGAAATAGGCTTTC-3’	21 mer	
MMP13 F	5’-TGCAGCTGTTCACTTTGAGG-3’	20 mer	138 bp
MMP13 R	5’-TCACCAATTCCTGGGAAGTCT-3’	21 mer	
MMP14 F	5’-TGATGCTGCTCTCTTCTGGA-3’	20 mer	105 bp
MMP14 R	5’-GGGTACTCGCTATCCACTGC-3’	20 mer	
SERPINE1 F	5’-GTGGACTTTTCAGAGGTGGAG-3’	21 mer	144 bp
SERPINE1 R	5’-GAAGTAGAGGGCATTCACCAG-3’	21 mer	
E-cadherin F	5’-GCAGTGACGAATGTGGTACC-3’	20 mer	182 bp
E-cadherin R	5’-GTGTCTGGCTCCTGGGCAGT-3’	20 mer	
N-cadherin F	5’-GAATTCAGCACCCGCCTCAG -3’	20 mer	172 bp
N-cadherin R	5’-GCTGCATATATCGATCTGGG-3’	20 mer	
TWIST F	5’-CTACGCCTTCTCGGTCTG-3’	18 mer	121 bp
TWIST R	5’-CTTCTCTGGAAACAATGACATCT-3’	23 mer	
SLUG F	5’-TTCACTCCGAAGCCAAATG-3’	19 mer	121 bp
SLUG R	5’-TCTCTCTGTGGGTGTGTG-3’	18 mer	
SNAIL F	5’-CCACAAGCACCAAGAGTC-3’	18 mer	136 bp
SNAIL R	5’-TGGCAGTGAGAAGGATGT-3’	18 mer	
ZEB1 F	5’-TGTGCCAATTTGTTCCTGTA-3’	20 mer	113 bp
ZEB1 R	5’-TGAGATGGGAGTCTGGTAAA-3’	20 mer	

Supplementary Table S2. Plasmids construction primer set

Primer name	Primer sequence	length
SERPINE1 OVER (*Hin*dIII)	5’-GATCAAGCTTACCATGCAGATGTCTCCAGCCCT-3’	33 mer
SERPINE1 OVER (*Xho* I)	5’-GATCCTCGAGCGGGGTTCCATCACTTGGCC-3’	30 mer
SERPINE1 promoter (*Nhe* I)	5’-GATCGCTAGCGCTGGCTGCTTAACCAAAAC-3’	30 mer
SERPINE1 promoter (*Hin*dIII)	5’-GATCAAGCTTTAGAGTCGCTGGGAAGCTGT-3’	30 mer
MMP1 promoter (*Kpn* I)	5’-GATCGGTACCCACACCTCCAACCTCAGCCA-3’	19 mer
MMP1 promoter (*Xho* I)	5’-GATCCTCGAGCAGGACTAGGCAGGTGAGGG-3’	21 mer

Supplementary Figure S1

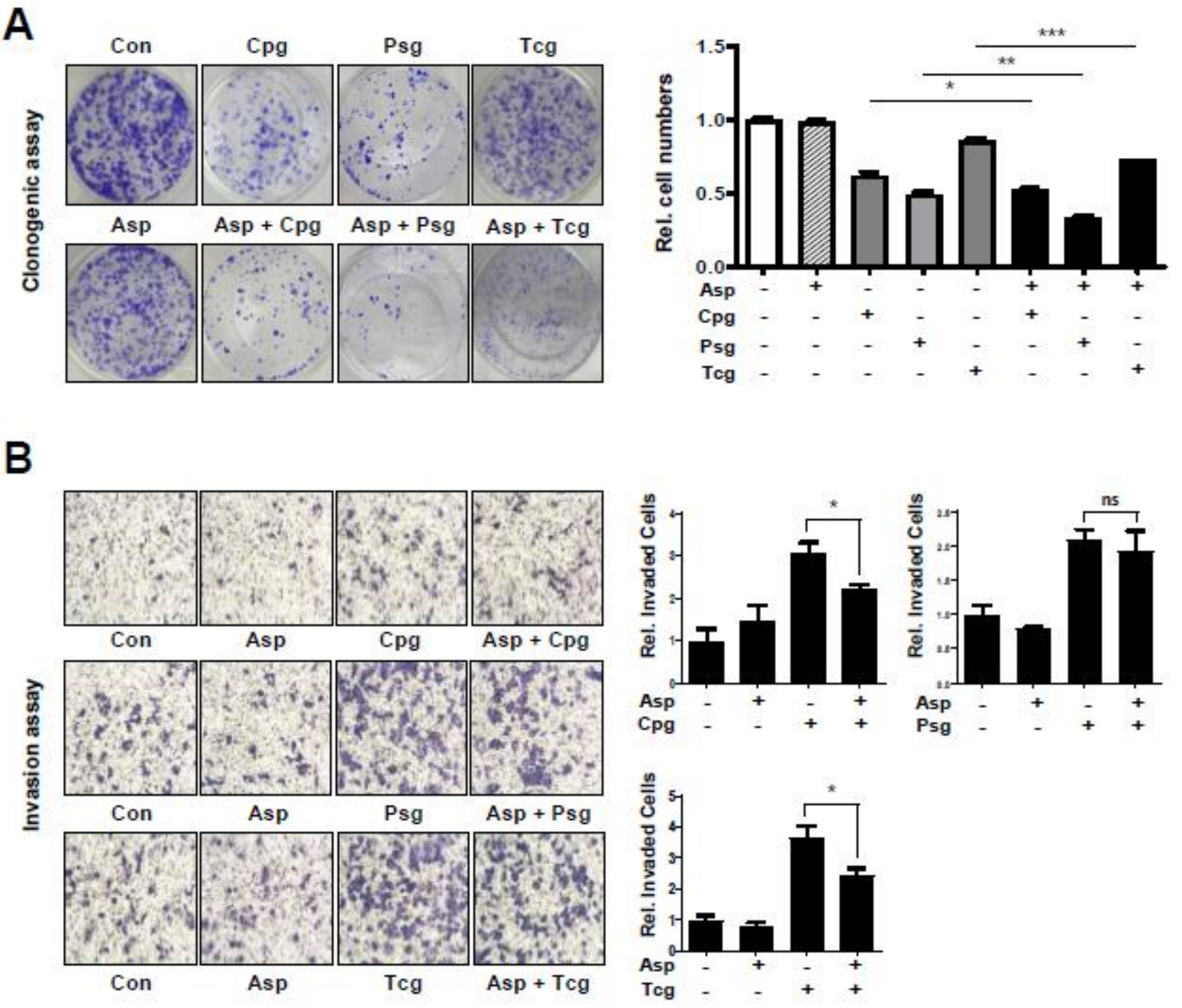

Figure S1. The synergistic effect of each purinergic antiplatelet agent and aspirin was investigated in HCT116 human colon cancer cells. Cells were treated with each of the purinergic antiplatelet agents (1000 μM Asp, 100 μM Cpg, 100 μM Psg and 20 μM Tcg) alone or in combination with Asp, followed by **(A)** cell proliferation and **(B)** invasion. Compared with the use of each purinergic antiplatelet agent alone, it was confirmed that when administered in combination with aspirin, it showed some inhibition of both cell proliferation and invasiveness. Statistical significance was determined using ordinary two-way ANOVA and student's *t*-tests, *ns*, not significant; *, $P < 0.05$; **, $P < 0.01$; ***, $P < 0.001$, $n \geq 3$ per group.

Supplementary Figure S2

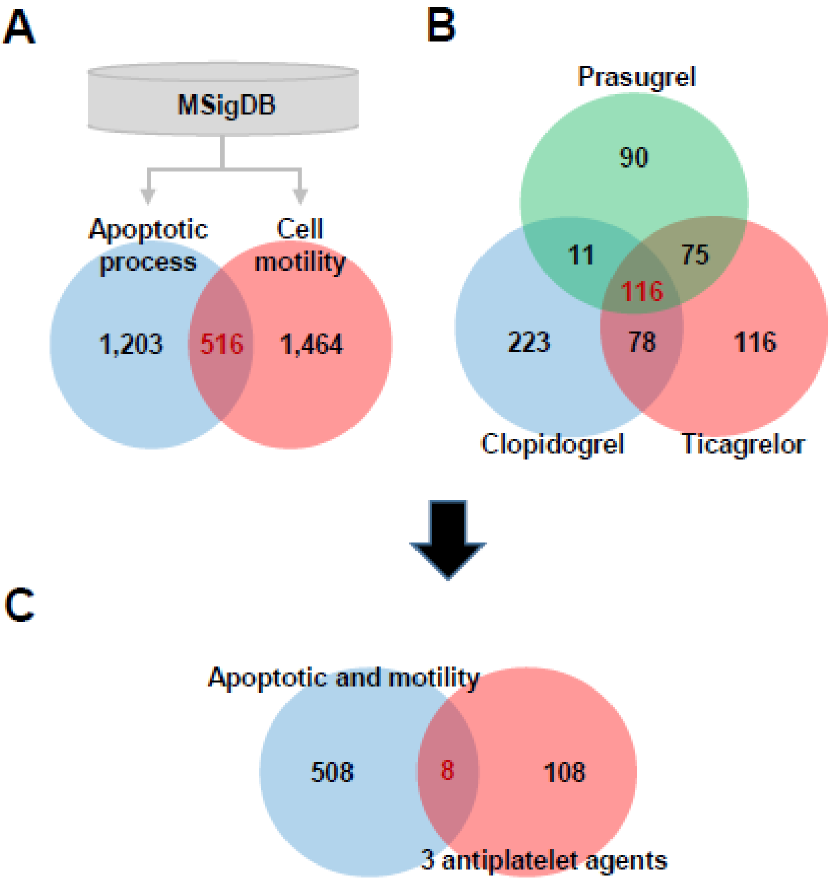

Overlapping genes related to apoptotic process, cell motility and 3 antiplatelet agents
ARHGEF2, BMP4, BTG1, DDIT4, FOXC1, SERPINE1, SH3KBP1, TNFRSF10B

Figure S2. Identification of commonly expressed candidate genes by comparing genes related to cell motility and apoptosis with genes co-expressed by treatment with 3 antiplatelet agents. (A) Common genes related to apoptotic process and cell motility in MSigDB. **(B)** Common genes related to treatment with 3 antiplatelet agents. Statistically significant common genes were selected using fold change and student's *t*-tests. (2-fold change and $P < 0.001$). **(C)** The eight overlapping genes associated with apoptotic process, cell motility, and 3 antiplatelet agents.

Supplementary Figure S3

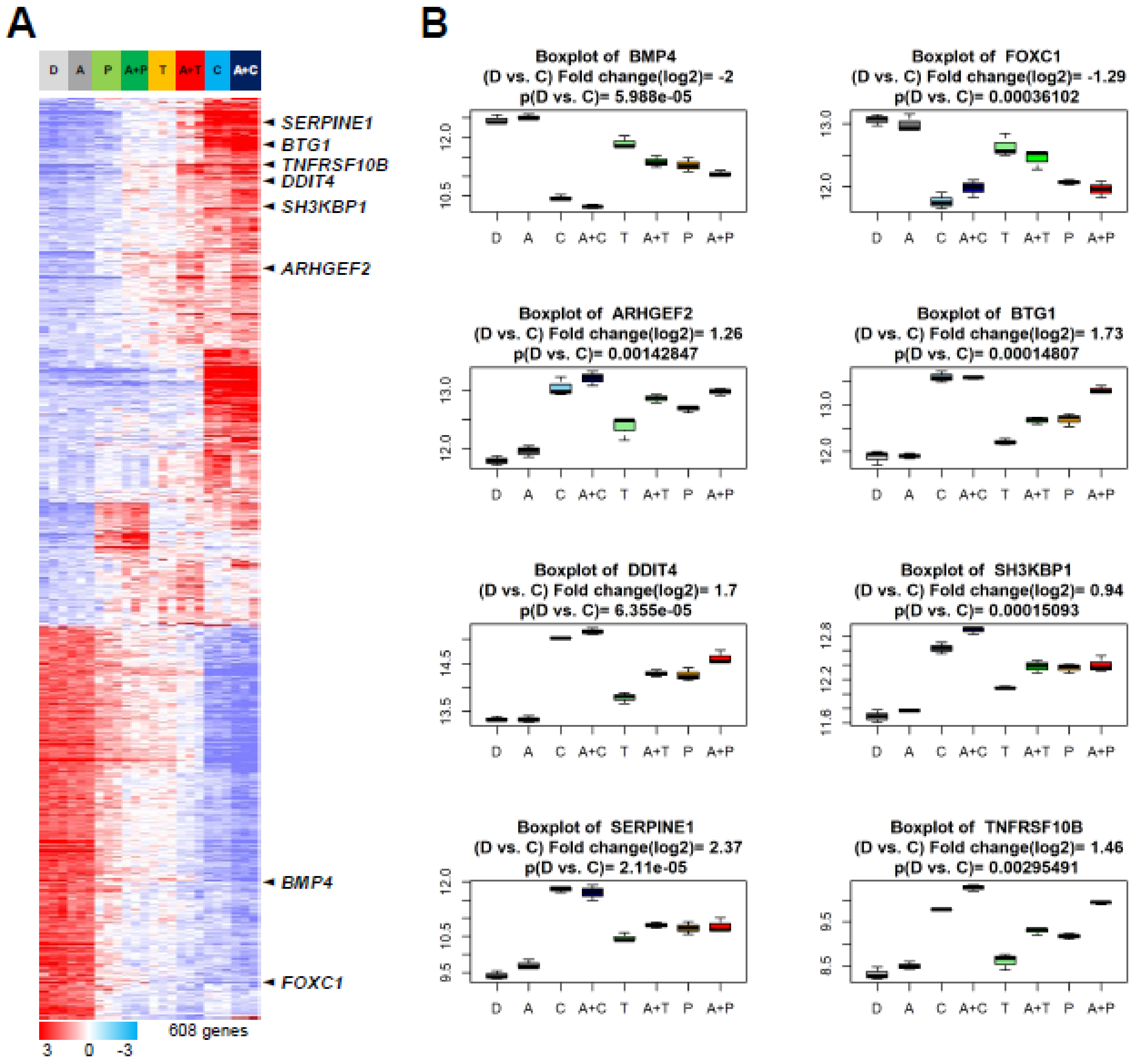

Figure S3. Expression patterns and boxplot of eight candidate genes. **(A)** Expression patterns of eight candidate genes were confirmed in heatmap of Figure 3A. D, DMSO and A, aspirin were control groups, and the samples treated with 3 antiplatelet agents (P, prasugrel; T, ticagrelor; C, clopidogrel) and were comparison groups (D and A vs. P and A+P, T and A+T, C and A+C). **(B)** Expression level of eight candidate genes were confirmed by boxplot in each sample. Fold change was calculated on a log2 scale and p-value were calculated by student's *t*-tests.

Supplementary Figure S4

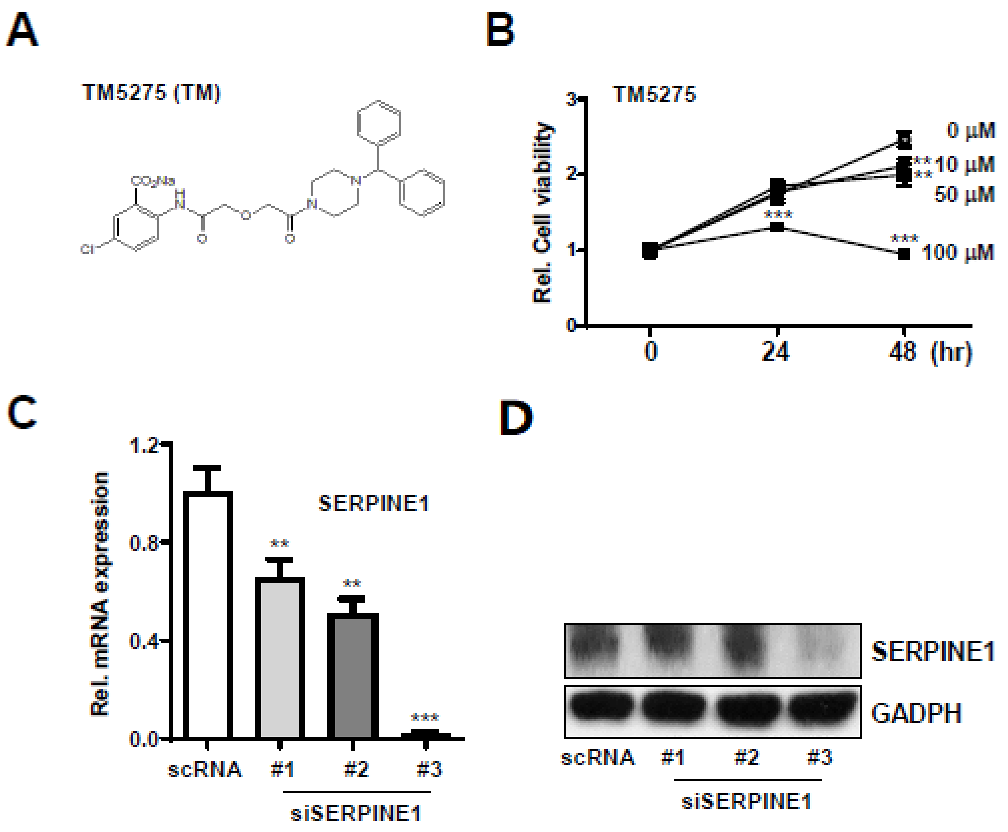

Figure S4. The effect of the reduction of SERPINE1 expression on cell invasion was confirmed. (A) Structure of TM5275, known as an inhibitor of SERPINE1. (B) Cell viability was determined by MTT assay for various concentrations of TM5275 (0, 10, 50 and 100 µM). (C and D), inhibition of SERPINE1 (C) mRNA and (D) protein expression was confirmed using three different siRNAs. Statistical significance was determined using ordinary two-way ANOVA and student's *t*-tests, **, $P < 0.01$; ***, $P < 0.001$, $n \geq 3$ per group.

A

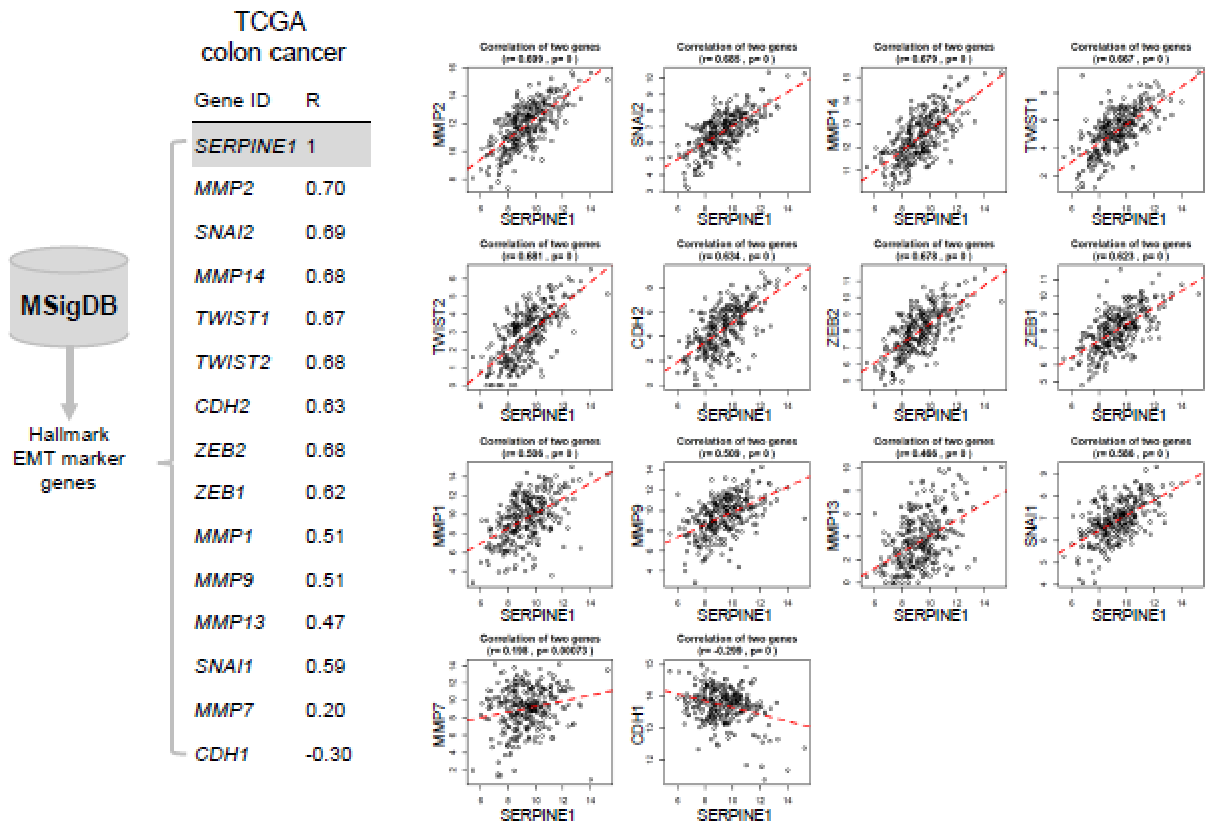
TCGA
colon cancer
Gene ID R
SERPINE1 1
MMP2 0.70
SNAI2 0.69
MMP14 0.68
TWIST1 0.67
TWIST2 0.68
CDH2 0.63
ZEB2 0.68
ZEB1 0.62
MMP1 0.51
MMP9 0.51
MMP13 0.47
SNAI1 0.59
MMP7 0.20
CDH1 -0.30
MSigDB
Hallmark
EMT marker
genes
MMP2
SNAI2
MMP14
TWIST1
TWIST2
CDH2
ZEB2
ZEB1
MMP1
MMP9
MMP13
SNAI1
MMP7
CDH1
SERPINE1

B

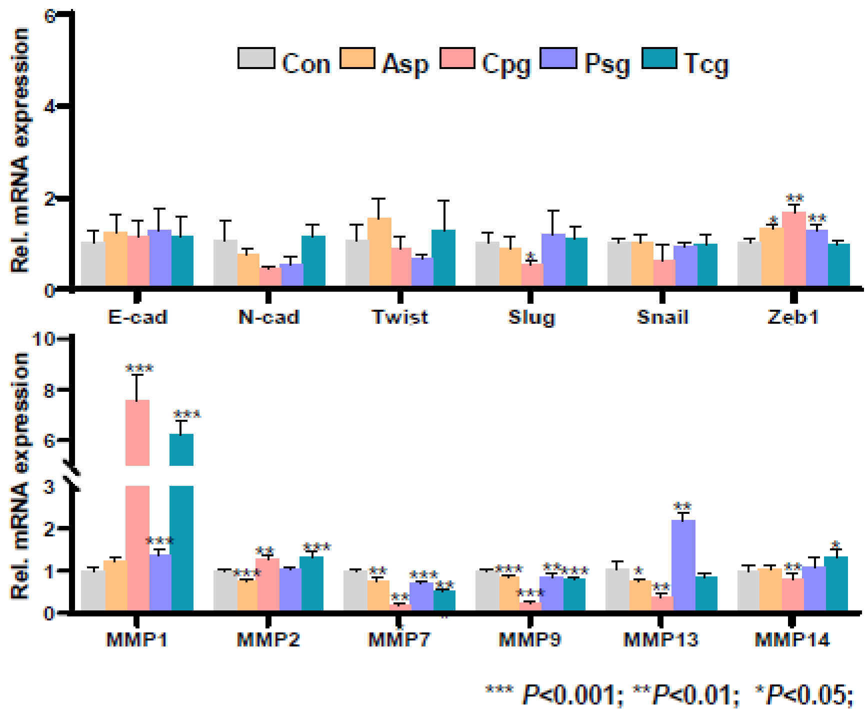
Con
Asp
Cpg
Psg
Tcg
Rel. mRNA expression
E-cad
N-cad
Twist
Slug
Snail
Zeb1
MMP1
MMP2
MMP7
MMP9
MMP13
MMP14
*** P<0.001; **P<0.01; *P<0.05;

Figure S5. Confirmation of association between SERPINE1 and EMT factor, MMP1. (A) Genes of the cancer Hallmark EMT marker in MSigDB were selected, and the correlation with the SERPINE1 was calculated in the TCGA colon cancer cohort. **(B)** MMP1, one of the EMT factors, increased by antiplatelet was identified (red box). Statistical significance was determined using ordinary two-way ANOVA and student's *t*-tests, *, $P < 0.05$; **, $P < 0.01$; ***, $P < 0.001$, $n \geq 3$ per group.

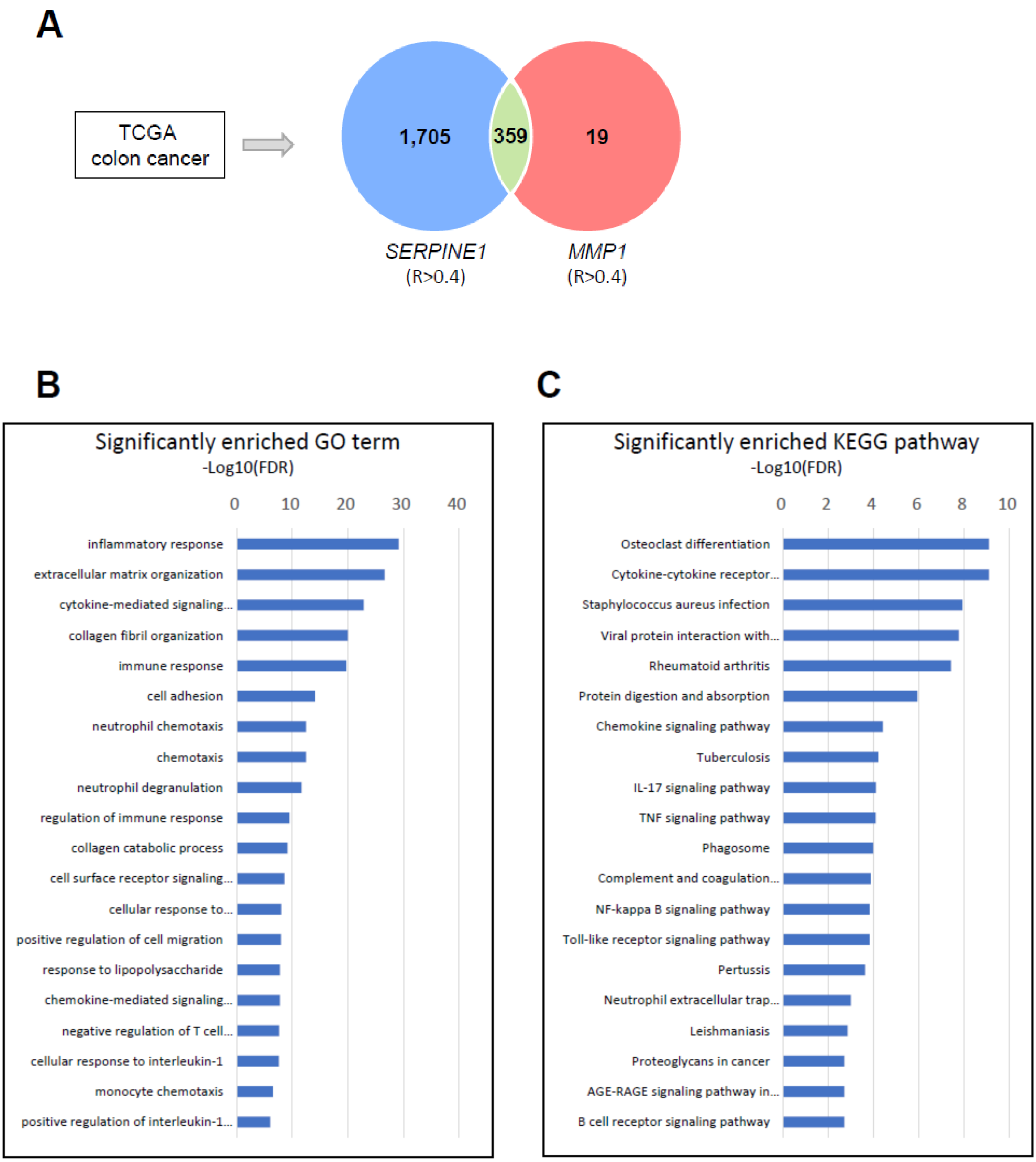

Figure S6. Confirmation and DAVID analysis of genes related to the expression pattern of SERPINE1 and MMP1. **(A)** Common genes to the expression pattern of SERPINE1 and MMP1 were identified in the TCGA colon cancer. **(B)** Gene Ontology and **(C)** KEGG pathway analysis were visualized based on FDR values.
